# Inhibition of HMGB1 reduces rat spinal cord astrocytic swelling and AQP4 expression after oxygen-glucose deprivation and reoxygenation via TLR4 and NF-κB signaling in an IL-6-dependent manner

**DOI:** 10.1186/s12974-017-1008-1

**Published:** 2017-11-25

**Authors:** Lin Sun, Man Li, Xun Ma, Haoyu Feng, Junlai Song, Cong Lv, Yajun He

**Affiliations:** 1 Department of Orthopedics, Shanxi Academy of Medical Sciences, Shanxi Da Yi Hospital, Shanxi Da Yi Hospital affiliated to Shanxi Medical University, Taiyuan, 030032 China; 2 Department of Neurology, Second Hospital of Shanxi Medical University, Shanxi Medical University, Taiyuan, 030001 China

**Keywords:** High mobility group box-1, Aquaporin-4, Toll-like receptor 4, Nuclear factor-kappa B, Interleukin-6, Astrocyte swelling, Spinal cord injury

## Abstract

**Background:**

Spinal cord astrocyte swelling is an important component to spinal cord edema and is associated with poor functional recovery as well as therapeutic resistance after spinal cord injury (SCI). High mobility group box-1 (HMGB1) is a mediator of inflammatory responses in the central nervous system and plays a critical role after SCI. Given this, we sought to identify both the role and underlying mechanisms of HMGB1 in cellular swelling and aquaporin 4 (AQP4) expression in cultured rat spinal cord astrocytes after oxygen-glucose deprivation/reoxygenation (OGD/R).

**Methods:**

The post-natal day 1–2 Sprague-Dawley rat spinal cord astrocytes were cultured in vitro, and the OGD/R model was induced. We first investigated the effects of OGD/R on spinal cord astrocytic swelling and HMGB1 and AQP4 expression, as well as HMGB1 release. We then studied the effects of HMGB1 inhibition on cellular swelling, HMGB1 and AQP4 expression, and HMGB1 release. The roles of both toll-like receptor 4 (TLR4)/nuclear factor-kappa B (NF-κB) signaling pathway and interleukin-6 (IL-6) in reducing cellular swelling resulting from HMGB1 inhibition in spinal cord astrocytes after OGD/R were studied. Intergroup data were compared using one-way analysis of variance (ANOVA) followed by Dunnett’s test.

**Results:**

The OGD/R increased spinal cord astrocytic swelling and HMGB1 and AQP4 expression, as well as HMGB1 release. Inhibition of HMGB1 using either HMGB1 shRNA or ethyl pyruvate resulted in reduced cellular volume, mitochondrial and endoplasmic reticulum swelling, and lysosome number and decreased upregulation of both HMGB1 and AQP4 in spinal cord astrocytes, as well as HMGB1 release. The HMGB1 effects on spinal cord astrocytic swelling and AQP4 upregulation after OGD/R were mediated—at least in part—via activation of TLR4, myeloid differentiation primary response gene 88 (MyD88), and NF-κB. These activation effects can be repressed by TLR4 inhibition using CLI-095 or C34, or by NF-κB inhibition using BAY 11-7082. Furthermore, either OGD/R or HMGB1 inhibition resulted in changes in IL-6 release. IL-6 was also shown to mediate AQP4 expression in spinal cord astrocytes.

**Conclusions:**

HMGB1 upregulates AQP4 expression and promotes cell swelling in cultured spinal cord astrocytes after OGD/R, which is mediated through HMGB1/TLR4/MyD88/NF-κB signaling and in an IL-6-dependent manner.

## Background

Spinal cord edema is a major, secondary injury resulting after spinal cord injury (SCI). It is closely associated with subsequent neurological deficits, as well as poor functional recovery and therapeutic resistance [1–3]. Given these long-term consequences, it is important to understand the mechanisms behind SCI-induced edema. A better mechanistic understanding will be very helpful for the development of treatment strategies and medical therapeutics for SCI [2, 4].

Astrocytes are the major glial cell type in the central nervous system (CNS). They undergo variable degrees of activation in response to all forms of CNS injury and disease, producing pro-inflammatory cytokines and accumulating most edematous fluid intracellularly [5–7]. Astrocytic swelling (cytotoxic edema) is an important, initial component of spinal cord edema and generates the driving force for the influx of ionic and vasogenic edema [8–10]. Aquaporin-4 (AQP4) is a water channel protein that has been found in astrocytes along the entirety of the spinal cord. With regards to its specific astrocytic localization, it is found not only in end-feet, but also dispersed in the cytoplasm of reactive astrocytes [11, 12]. AQP4 facilitates transmembrane water movement in the CNS, and experimental evidence has demonstrated that AQP4 expression is associated with spinal cord edema following SCI [13, 14].

Inflammation after SCI is a key mechanism mediating later secondary spinal cord injury and is largely initiated when local cells on the site of injury (e.g., microglia, astrocytes) secrete injury-associated molecules [15, 16]. The high mobility group box-1 (HMGB1) is a highly conserved, non-histone DNA-binding protein that has been recently recognized as a key mediator in inflammation and neuroinflammation [15, 17]. It can be actively secreted from reactive astrocytes and microglia in the CNS as well as passively released from necrotic cells after pathogenic insult or tissue injury [17–21]. Extracellular HMGB1 triggers inflammatory responses through the activation of multiple receptors, such as receptor for toll-like receptor-4 (TLR4), TLR2, and advanced glycation end product (RAGE) in immune-competent cells, neurons, and astrocytes [17, 22, 23]. HMGB1 signaling through these receptors promotes activation of the nuclear factor-kappa B (NF-κB) transcription factor, which is ubiquitously expressed and required for the expression of the many mediators of inflammatory responses and cell survival, such as TNF-α, interleukin-1β (IL-1β), and interleukin-6 (IL-6) [24–26]. HMGB1 and its signaling are associated with astrocytic swelling and the injury in the CNS. In the brain, HMGB1 and its signaling resulted in significantly increased astrocytic swelling as well as AQP4 expression after traumatic brain injury, cerebral ischemia, and hepatic encephalopathy. Critically, inhibition of such signaling blocked astrocytic swelling [27–31]. After SCI, increased expression of HMGB1 was observed in both humans and a rodent model of SCI [15, 32]. This converging evidence indicates that HMGB1 may play an amplifying role in tissue pathology and inflammation, leading to secondary damage after the initial spinal cord injury [15, 32, 33]. To date, the relationship between HMGB1 and spinal cord astrocytic swelling remains poorly understood, as does its potential regulatory mechanism(s).

Here, we sought to study (1) the role of HMGB1 in spinal cord astrocytic swelling and (2) AQP4 expression induced by oxygen-glucose deprivation/reoxygenation (OGD/R) injury. We then sought to elucidate the underlying molecular mechanisms for both. To this end, we first investigated HMGB1 expression and its ability to promote astrocytic swelling in cultured cells obtained from rat spinal cord subjected to OGD/R. We then studied effects of HMGB1 inhibition using either shRNA or ethyl pyruvate (EP) [34] on spinal cord astrocytic swelling and AQP4 expression. Since several potential cellular pathways could mediate the pro-inflammatory effects of HMGB1, we started our approach with the TLR4/NF-κB signaling pathway and its product, IL-6. This signaling pathway was chosen because past work reported its importance in the regulation of CNS astrocytic swelling and tissue edema in an HMGB1 challenge [27–30, 35, 36]. We found that HMGB1 inhibition reduced both spinal cord astrocytic swelling and aquaporin-4 expression after OGD/R in vitro. Importantly, these effects were regulated, in part, through TLR4/NF-κB signaling as well as IL-6.

## Methods

### Astrocyte culture

All experimental procedures in this study were approved by the Animal Care and Use Committee of Shanxi Medical University. All animals were treated in accordance with the guidelines provided in the National Institutes of Health (NIH) Guide for the Care and Use of Laboratory Animals. Primary spinal cord astrocyte cultures were prepared from post-natal day (PND) 1–2 Sprague-Dawley rats (Shanxi Medical University, RRID:RGD_5508397, Taiyuan, China) as previously described with minor modifications [37]. Briefly, rats were anesthetized with diethyl ether and then rapidly decapitated. Spinal cords were manually freed of meninges, cut into small pieces (approximately 0.125 mm^3^), and treated with 0.125% trypsin (Boster, Cat# PYG0015, Wuhan, China). The resulting mixture was passed through sterile nylon sieves and placed in Dulbecco’s modified Eagle medium (DMEM; Gibco, Life Technologies, Cat# C11995500BT, Grand Island, NY, USA) containing 10% fetal bovine serum (FBS; Gibco, Life Technologies, Cat# 16000-044). Cultures were incubated with 5% CO_2_ and 95% air at 37 °C. All culture media were changed twice weekly. After 4–5 days in culture, flasks were shaken at 200 rpm overnight at 37 °C to remove microglial cells and oligodendrocytes. To yield a higher percentage of spinal cord astrocytes, subculture was performed when cultured astrocytes were confluent (approximately 10 days after primary culture). Astrocytic purity was confirmed by staining with S100βprotein [37, 38]. These secondary cultured spinal cord astrocytes were used in all the subsequent experiments.

### Special chemicals and antibodies

Special chemicals include ethyl pyruvate (EP, Sigma-Aldrich Co., Cat# E47808, Saint Louis, MO, USA), CLI-095 (Invivo Gen, Cat# tlrl-cli95, San Diego, CA, USA), C34 (TOCRIS, Cat# 5373, Bristol, UK), recombinant HMGB1 (rHMGB1, ProSpec, Cat# pro-581-b, Rehovot, Israel), BAY 11-7082 (Sigma-Aldrich Co., Cat# B5556), IL-6 (PeproTech Inc., Cat# 400-06-2UG, Rocky Hill, NJ, USA), and neutralizing anti-rat-IL-6 antibody (R&D SYSTEMS, Cat# AF506, Minneapolis, MN, USA).

Primary antibodies include rabbit polyclonal anti-HMGB1 antibody for rat, mouse, and human (Abcam, Cat# ab18256, RRID:AB_444360, Cambridge, UK); rabbit polyclonal anti-AQP4 antibody for rat, mouse, human, and pig (Abcam, Cat# ab46182, RRID: AB_955676); mouse monoclonal anti-TLR4 antibody for rat, mouse, human, pig, baboon, bovine, and Chinese hamster (Novus, Cat# 76B357.1, RRID: AB_839000, Littleton, CO, USA); rabbit polyclonal anti-TLR4 antibody for rat, mouse, human, and rabbit (Boster, Cat# BA1717, RRID:AB_2716293); rabbit polyclonal anti-myeloid differentiation primary response gene 88 (MyD88) antibody for rat and human (Abcam, Cat# ab131071, RRID: AB_11156885); mouse monoclonal anti-IκBα antibody for rat, mouse, human, monkey, bovine, pig, and guinea pig (Cell Signaling Technology, Cat# 4814, RRID: AB_390781, Boston, MA, USA); mouse monoclonal anti-p-IκBα antibody for rat, mouse, human, and monkey (Cell Signaling Technology, Cat# 9246, RRID:AB_2267145); rabbit monoclonal anti-NF-κB antibody for rat, mouse, human, monkey, and bovine (Cell Signaling Technology, Cat# 4764, RRID:AB_823578); mouse monoclonal anti-GAPDH antibody for rat, mouse, and human (Beyotime, Cat# AF0006, RRID: AB_2715590, Shanghai, China); mouse monoclonal anti-Histone H3 antibody for rat, mouse, and human (Beyotime, Cat# AF0009, RRID: AB_2715593); and mouse monoclonal anti-S100β antibody for rat, mouse, human, rabbit, and pig (Boster, Cat# BM0120, RRID:AB_2716291).

Secondary antibodies include horseradish peroxidase-conjugated goat anti-rabbit secondary antibody (absin, Cat# abs20002A, RRID: AB 2716554, Shanghai, China), horseradish peroxidase-conjugated goat anti-mouse secondary antibody (absin, Cat# abs20001A, RRID: AB 2716555), Cy3-conjugated goat anti-rabbit secondary antibody (Boster, Cat# BA1032, RRID: AB_2716305), and FITC-conjugated goat anti-mouse secondary antibody (ZSGB-BIO, Cat# BA1032, Beijing, China, RRID: AB_2716306).

### Experimental protocol

#### Experiment 1. Effects of OGD/R on cellular swelling, HMGB1 and AQP4 expression in spinal cord astrocytes, and HMGB1 and IL-6 levels in the surrounding medium

Oxygen-glucose deprivation and reoxygenation (OGD/R) injury were induced in cultured spinal cord astrocytes. Astrocytic volume as well as HMGB1 and AQP4 expression was subsequently measured at 2, 6, 12, 24, and 48 h during the reoxygenation process after OGD. Enzyme-linked immunosorbent assay (ELISA) was then used to measure HMGB1 and IL-6 levels in the medium at 6, 12, and 24 h during reoxygenation.

#### Experiment 2. Effects of HMGB1 inhibition on cellular swelling as well as HMGB1, AQP4, and TLR4 expression in spinal cord astrocytes and HMGB1 and IL-6 levels in the surrounding medium after OGD/R

The experimental groups consisted of the following: normal, OGD/R, OGD/R + HMGB1 shRNA, OGD/R + non-targeting shRNA, and OGD/R + EP (HMGB1 inhibitor, 12 μM). With the exception of the normal group, all other group measurements were performed at 6, 12, and 24 h during the reoxygenation process after OGD. Measurements included astrocytic volume and astrocytic morphology and ultrastructure, as well as HMGB1, AQP4, and TLR4 expression. ELISA was used to determine HMGB1 and IL-6 levels in the surrounding medium.

#### Experiment 3. Role of TLR4/NF-κB signaling pathway in reducing cellular swelling resulting from HMGB1 inhibition in spinal cord astrocytes after OGD/R

To investigate the role of TLR4, spinal cord astrocytes were randomly divided into the following groups: normal, OGD/R, OGD/R + HMGB1 shRNA, OGD/R + non-targeting shRNA, OGD/R + CLI-095 (TLR4 inhibitor, 5 μM), OGD/R + C34 (another TLR4 inhibitor, 15 μM), OGD/R + HMGB1 shRNA + rHMGB1 (10 ng/ml), and OGD/R + EP. The astrocytic volume and the expression levels of TLR4, MyD88, IκBα, p-IκBα, and AQP4 were measured. Nuclear expression levels of NF-κB were also measured, in addition to IL-6 levels in the surrounding medium. Measurements were obtained after undergoing reoxygenation for 24 h after OGD.

To investigate the role of NF-κB, spinal cord astrocytes were randomly divided into the following groups: normal, OGD/R, OGD/R + HMGB1 shRNA, OGD/R + non-targeting shRNA, OGD/R + BAY 11-7082 (NF-κB inhibitor, 5 μM), and OGD/R + EP. The astrocytic volume as well as expression levels of IκBα, p-IκBα, and AQP4 was measured. In addition, expression level of nuclear NF-κB was measured and IL-6 levels in the surrounding medium were measured using ELISA. Measurements were obtained after undergoing reoxygenation for 24 h after OGD.

#### Experiment 4. Effects of rHMGB1 and IL-6 on regulating AQP4 expression in spinal cord astrocytes

To investigate the role of rHMGB1, spinal cord astrocytes were exposed to rHMGB1 at a series of concentrations (0, 0.1, 1, 10, or 20 ng/ml). After 24 h of exposure, AQP4 expression was measured using Western blot analysis.

We next investigated the role of IL-6 on regulating AQP4 expression in spinal cord astrocytes. First, spinal cord astrocytes were randomly divided into four groups, in which spinal cord astrocytes were exposed to IL-6 (0, 0.1, 1, or 10 ng/ml) for 24 h, and AQP4 expression was measured using Western blot. Second, spinal cord astrocytes were exposed to different astrocyte conditioned media (ACM), which originated from the astrocyte cultures in the OGD/R, OGD/R + HMGB1 shRNA, and OGD/R + non-targeting shRNA groups in Experiment 2. All ACM were harvested after undergoing reoxygenation for 24 h after OGD. The resulting AQP4 expression in spinal cord astrocytes was measured using Western blot. Finally, the neutralizing anti-rat-IL-6 antibody was used to reverse the effect of IL-6 on AQP4 expression in cultured spinal cord astrocytes. Spinal cord astrocytes were randomly divided into the following groups: normal, astrocytes + IL-6 (0.15 ng/ml), astrocytes + IL-6 (0.15 ng/ml) + anti-IL6 antibody (0.1 μg/ml), astrocytes + OGD6h/R24h ACM, and astrocytes + OGD6h/R24h ACM + anti-IL6 antibody (0.1 μg/ml). After 24 h exposure, AQP4 expression was measured using Western blot.

All experiments were repeated at least three times, and the average values were shown. In the normal group of all the experiments, spinal cord astrocytes were cultured in DMEM containing 10% FBS and incubated with 5% CO_2_ and 95% air at 37 °C. All chemical inhibitors were dissolved in the medium without additional organic solvents. Chemical concentrations and durations used in all experiments were chosen on pilot experiments conducted in our lab.

### OGD/R procedure

Cultured spinal cord astrocytes were washed three times with phosphate-buffered saline (PBS) and incubated in serum-free DMEM without glucose (Gibco, Life Technologies, Cat# 11966025). Astrocytes were then placed in an anaerobic chamber filled with 1% O_2_, 5% CO_2_, and 94% N_2_ at 37 °C for 6 h (oxygen-glucose deprivation, OGD). After 6 h of OGD exposure, astrocytes were rinsed once with PBS and then incubated under normal conditions (reoxygenation). Different inhibitors were added into the medium prior to OGD injury and at the time of reoxygenation.

### shRNA-mediated HMGB1 knockdown

An HMGB1 shRNA lentiviral vector was synthesized by HANBIO Company (Shanghai, China). The targeting sequence was as follows: HMGB1 (Gene ID: 3146, shRNA sequence: 5′-GGCAAAGGCTGACAAGGCTCGTTATTTCAAGAGAATAACGAGCCTTGTCAGCCTTTGCCTTTTTT-3′). A non-targeting shRNA (also purchased from HANBIO) was used as a negative control for all experiments. Either HMGB1 shRNA or the non-targeting shRNA lentiviral vector was introduced into cultures using DMEM and at a final multiplicity of infection (MOI) of 60. Spinal cord astrocytes were incubated with viral vectors for 12 h, and then, the medium was changed to normal medium. Astrocytes were then cultured for additional 72 h under normal conditions before OGD/R. The effective shRNA sequence for the three candidate target sequences was designed by HANBIO Company. The transfection MOI for the HMGB1 shRNA lentiviral vector was determined based on pilot experiments. The shRNA knockdown efficiency was evaluated using Western blot.

### Astrocytic volume analysis

Astrocyte volume analysis was performed using a Live Cell Imaging System (DeltaVision Applied Precision, GE Healthcare, Chicago, IL, USA) as previously described with some modifications [39, 40]. Cultured spinal cord astrocytes were plated on cover glass-bottomed 35-mm dishes. Just prior to cell volume measurement, astrocytes were stained with 10 μM 1,1′-dioctadecyl-3,3,3′,3′-tetramethylindocarbocyanine perchlorate (Dil, Beyotime, Cat# C1036) under normal conditions for 1 h. After being washed with DMEM, astrocytes were dissociated into single cells and suspended in DMEM containing FBS. Immediately after, astrocytes were scanned using the Live Cell Imaging System. Cells were excited at 549 nm, and emission data were collected at 565 nm. Z-stack image series of single spinal cord astrocytes were acquired in accordance to manual tracing of the cell perimeter using a step size of 2 μm. A compiled Z-slice cellular image was obtained by overlapping each slice in the stack. This compiled image was then used for volumetric calculations for that particular cell. The average value of the four measured diameters of the largest compiled Z-slice image was defined as the cellular diameter. Cellular volume was calculated as follows: Volume = 4/3 × π × *r* ^3^ (where *r* is radius). At least 15 cells were analyzed for each group from at least three separate experiments. The average values were used in analysis.

### Cellular morphological and ultrastructural characterization

Transmission electron microscopy was used to characterize cellular morphology and ultrastructural features of spinal cord astrocytes. Cultured astrocytes were rinsed in PBS and fixed with 0.1 M cacodylate-buffered glutaraldehyde for 1 h. After being washed in cacodylate buffer for 10 min, astrocytes were fixed with 1% osmium tetroxide in cacodylate buffer for 2 h. Astrocytes were then dehydrated in a series of alcohol concentrations, and then, they were embedded in Epon. Thin sections (50 nm) were made and stained using uranyl acetate and lead citrate. Changes in astrocytic swelling as well as mitochondrial, endoplasmic reticulum, and lysosomal alterations were subsequently observed using transmission electron microscopy (JEM-1011, Tokyo, Japan).

### Western blot

Successive preparations of the plasma membrane and cytoplasmic extracts and nuclear extracts were made using a commercially available protein extraction kit (Beyotime, Cat# P0033) according to the manufacturer’s instructions. Briefly, cultured spinal cord astrocytes were washed with ice-cold PBS, harvested using a cell scraper, and centrifuged at 3000×*g* for 5 min. Cell pellets were then resuspended in a membrane and cytoplasmic extraction reagent containing phenylmethanesulfonyl fluoride (PMSF), phosphatase inhibitors, and protease inhibitors; vortexed for 5 s; and incubated on ice for 30 min. Lysates were then centrifuged at 12000×*g* at 4 °C for 10 min to obtain membrane-bound and cytoplasmic protein fractions for later expression analysis, with the exception of NF-κB. For NF-κB analysis, cell pellets were resuspended in a nuclear extraction reagent containing PMSF, phosphatase inhibitors, and protease inhibitors; vortexed for 5 s; and incubated on ice for 30 min. Lysates were centrifuged at 12000×*g* at 4 °C for 10 min to obtain nuclear protein fraction for NF-κB expression analysis. Prior to Western blot, all protein concentrations were determined using a BCA Protein Assay Kit (Beyotime, Cat# P0012S).

Protein (20 μg per lane) was subjected to electrophoresis using 10% sodium dodecyl sulfate polyacrylamide gels, followed by transfer to a polyvinylidene fluoride membrane (Millipore Corp. Billerica, MA, USA). After transfer, the membrane was blocked in 5% non-fat milk at 37 °C for 2 h. One of the following primary antibodies was then added: anti-HMGB1 (1:1000, Abcam), anti-AQP4 (1:800, Abcam), anti-TLR4 (1:800, Novus), anti-MyD88 (1:800, Abcam), anti-IκBα (1:1000, Cell Signaling Technology), anti-p-IκBα (1:1000, Cell Signaling Technology), anti-NF-κB (1:1000, Cell Signaling Technology), anti-GAPDH (1:1000, Beyotime), or anti-Histone H3 (1:1000, Beyotime). All primary antibodies were incubated at 4 °C overnight. After being washed by PBS containing Tween-20 (PBST), membranes were incubated with horseradish peroxidase-conjugated goat anti-rabbit secondary antibody (1:5000, absin) or horseradish peroxidase-conjugated goat anti-mouse secondary antibody (1:5000, absin) at room temperature for 2 h. Bands were visualized using enhanced chemiluminescence (ECL, Beyotime, Cat# P0018) and subsequently analyzed using Quantity One software (Bio-Rad, http://www.bio-rad.com, RRID: SCR_014280). The expression levels of proteins in the plasma membrane and cytoplasmic extracts were normalized to that of GAPDH. The expression levels of NF-κB in the nuclear extracts were normalized to that of Histone H3. The average band density for the normal group was set at 1.0, and all other band density values were normalized by the average value of the normal group.

### Immunofluorescence

Cultured spinal cord astrocytic purity was confirmed by staining with S100β protein using immunofluorescence. The levels of HMGB1, AQP4, TLR4, and NF-κB in Experiments 2 and 3 at 24 h during reoxygenation process after OGD were evaluated by immunofluorescence. Cultured spinal cord astrocytes on coverslips were rinsed with PBS and fixed with 4% paraformaldehyde at 4 °C for 20 min. Cells were then permeabilized with 0.1% Triton X-100 at room temperature for 10 min and blocked with 5% goat serum (Solarbio, Cat# SL038, Beijing, China) at 37 °C for 1 h. Cells were then incubated with one of the following primary antibodies: anti-S100β (1:200, Boster), anti-HMGB1 (1:200, Abcam), anti-AQP4 (1:200, Abcam), anti-TLR4 (1:150, Bostor), or anti-NF-κB (1:100, Cell Signaling Technology) at 4 °C overnight. After being washed with PBS, cells were incubated with Cy3-conjugated goat anti-rabbit secondary antibody (1:800, Boster) or FITC-conjugated goat anti- mouse secondary antibody (1:1000, ZSGB-BIO) at 37 °C for 90 min. Cells were then counterstained with ProLong gold anti-fade reagent (Boster, Cat# AR1176) with DAPI for 2 h. Immunostaining was observed using fluorescence microscopy (OLYMPUS, Tokyo, Japan). Resulting image analysis was conducted using Image Pro plus 6.0 software (Media Cybernetics, http://image-pro-plus.updatestar.com), and the mean optical density values for each immunoreactive protein were measured.

### Enzyme-linked immunosorbent assay (ELISA)

The levels of HMGB1 and IL-6 released from cultured spinal cord astrocytes into the surrounding medium were measured by ELISA. Cultured media were sampled. HMGB1 and IL-6 concentrations were then determined using a commercially available ELISA kit (Xitang, HMGB1, Cat# F15640; IL-6, Cat# F15870, Shanghai, China) according to the manufacturer’s instructions. Absorbance was measured at 450 nm using a microplate reader.

### Statistical analysis

Three repeated tests were performed for each set of measurements, and the resulting data were expressed as the mean ± standard deviation (SD). The common statistical package SPSS 18.0 (IBM, https://www.ibm.com) was used for all statistical analysis. There were no repeated-measure (matched) data in our study. All data were analyzed using one-way analysis of variance (ANOVA) followed by Dunnett’s test. Values of *P* < 0.05 were considered statistically significant.

## Results

### Spinal cord astrocyte identification and HMGB1 knockdown

**Fig. 1:**
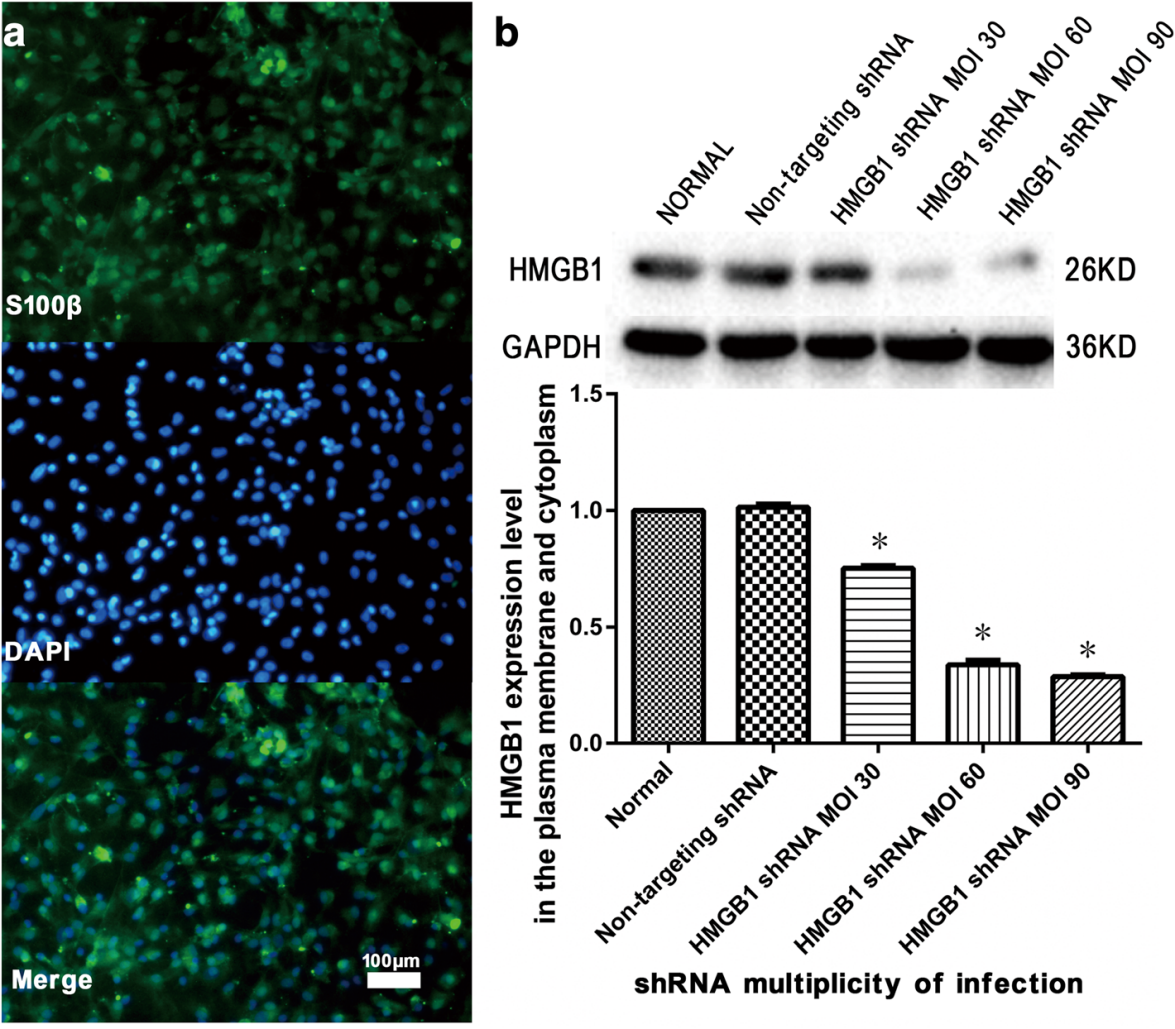
Spinal cord astrocyte identification and high mobility group box-1 (HMGB1) knockdown. **a** Spinal cord astrocytes were identified using immunofluorescence. The percentage of cells stained with the astrocytic marker S100β, which were identified as astrocytes, was more than 95% of the total cells (three replicates). **b** HMGB1 knockdown efficiency in the plasma membrane and cytoplasm of spinal cord astrocytes was evaluated using Western blot for HMGB1 protein levels. Results were obtained after 72 h of specific HMGB1 shRNA treatment. HMGB1 protein levels were decreased to approximately 30% of normal levels with shRNA multiplicity of infection 60 as compared to normal astrocytes. *P < 0.05 vs. normal group (three replicates)

Using the rat spinal cord astrocyte culture system, the percentage of cells stained with the astrocytic marker S100β, which was identified as astrocytes, was more than 95% of total cells (Fig. 1a). Astrocytic HMGB1 knockdown efficiency was evaluated using HMGB1 protein expression in both plasma membrane and cytoplasmic extracts using Western blot. Results showed that HMGB1 protein levels were markedly decreased to approximately 30% when compared with normal astrocytes. These levels were measured after 72 h of specifically targeted HMGB1 shRNA treatment with MOI 60 (*P* < 0.05, Fig. 1b).

### OGD/R increases cell swelling and HMGB1 and AQP4 expression in cultured spinal cord astrocytes as well as HMGB1 and IL-6 released into the surrounding medium

**Fig. 2:**
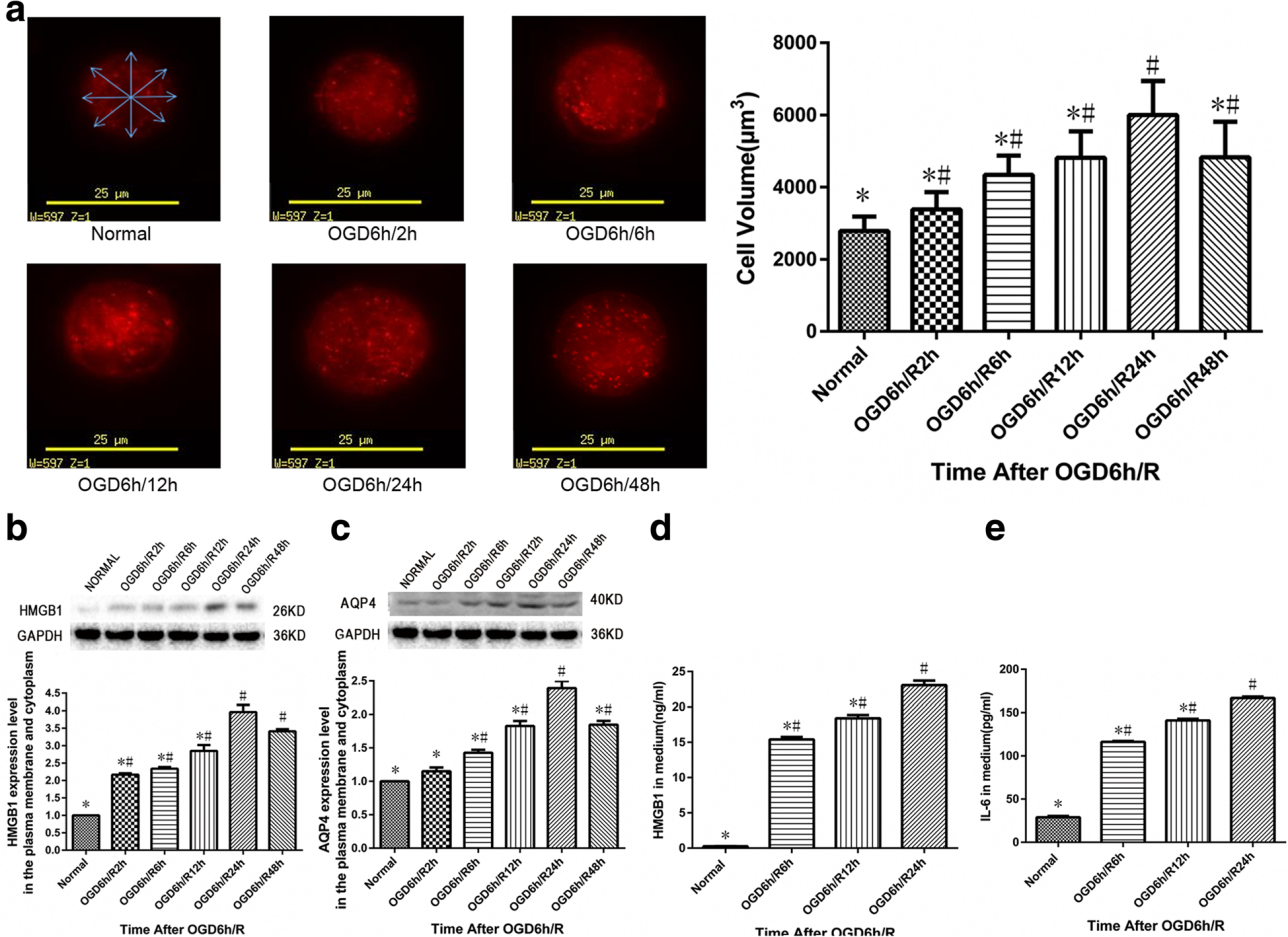
Effects of oxygen-glucose deprivation/reoxygenation (OGD/R) on cellular swelling, high mobility group box-1 (HMGB1), and aquaporin-4 (AQP4) expression in cultured spinal cord astrocytes as well as levels of HMGB1 and interleukin-6 (IL-6) released into the surrounding medium. **a** Astrocyte volume measurement was performed using a Live Cell Imaging System. Cellular volume was calculated by the average value of four measured diameters of the largest compiled Z-slice image. Cellular volumes of spinal cord astrocytes were significantly increased at 2, 6, 12, 24, and 48 h during reoxygenation after OGD when compared with normal astrocytes. #*P* < 0.05 vs. normal group; *P < 0.05 vs. OGD6h/R24h group (three replicates). **b** Membrane and cytoplasmic HMGB1 expression was significantly increased in spinal cord astrocytes at different time points after OGD/R. #*P* < 0.05 vs. normal group; *P < 0.05 vs. OGD6h/R24h group (three replicates). **c** Membrane and cytoplasmic AQP4 expression was significantly increased in spinal cord astrocytes at different time points after OGD/R. #*P* < 0.05 vs. normal group; *P < 0.05 vs. OGD6h/R24h group (three replicates). **d** HMGB1 levels in the surrounding medium of spinal cord astrocytes were significantly increased at 6, 12, and 24 h during reoxygenation after OGD/R. #*P* < 0.05 vs. normal group; *P < 0.05 vs. OGD6h/R24h group (three replicates). **e** IL-6 levels in the surrounding medium of spinal cord astrocytes were significantly increased at 6, 12, and 24 h during reoxygenation after OGD/R. #*P* < 0.05 vs. normal group; *P < 0.05 vs. OGD6h/R24h group (three replicates)

Cellular volumes were measured to determine the effects of OGD/R on spinal cord astrocytic swelling. When compared with normal astrocytes, astrocytic cellular volumes after OGD/R were significantly increased at 2, 6, 12, 24, and 48 h during reoxygenation (*P* < 0.05, Fig. 2a). The maximum increase was observed at 24 h during the reoxygenation process (*P* < 0.05).

Plasma membrane and cytoplasmic extracts were isolated from cultured spinal cord astrocytes at different time points after OGD/R and analyzed for HMGB1 and AQP4 expression using Western blot. A significant increase in HMGB1 expression was first observed at 2 h during reoxygenation. This increase in expression remained elevated for up to 48 h when compared with normal astrocytes (*P* < 0.05, Fig. 2b). Some AQP4 expression was seen in normal astrocytes, but AQP4 protein was found to have significantly increased during the reoxygenation period, reaching a peak at 24 h during reoxygenation (*P* < 0.05, Fig. 2c).

ELISA was used to examine HMGB1 levels and IL-6 released from cultured spinal cord astrocytes into the surrounding medium at 6, 12, and 24 h during the reoxygenation process after OGD. As shown in Fig. 2d, e, OGD/R increased the release of both HMGB1 and IL-6 into the extracellular space (*P* < 0.05).

### Inhibiting HMGB1 reduces cellular swelling in cultured spinal cord astrocytes after OGD/R

**Fig. 3:**
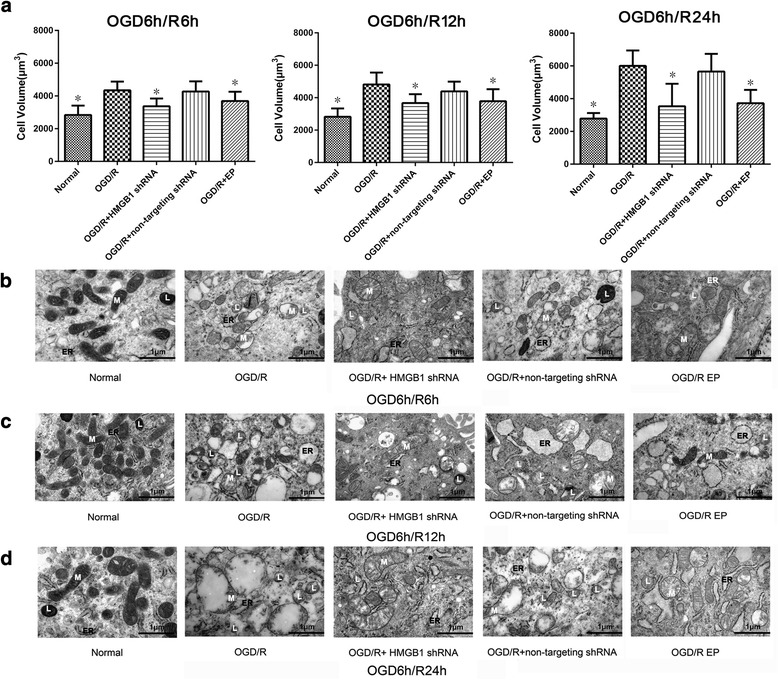
Effects of inhibiting high mobility group box-1 (HMGB1) on cellular swelling in cultured spinal cord astrocytes after oxygen-glucose deprivation/reoxygenation (OGD/R). **a** Astrocyte volume analysis was performed using a Live Cell Imaging System, and cellular volume was calculated by the average value of four measured diameters. Inhibiting HMGB1 using either HMGB1 shRNA or ethyl pyruvate (EP) significantly blocked increases in cellular volume of spinal cord astrocytes at 6, 12, and 24 h during reoxygenation when compared with astrocytes of the OGD/R group. *P < 0.05 vs. OGD/R group (three replicates). **b**—**d** Effects of inhibiting HMGB1 on spinal cord astrocytic morphology and ultrastructure were evaluated using transmission electron microscopy at 6, 12, and 24 h during reoxygenation after OGD. After OGD/R, spinal cord astrocytes showed swelling at 6, 12, and 24 h during reoxygenation. The mitochondrial (M) swelling, endoplasmic reticulum (ER) swelling and fragmentation, and an increase in the number of lysosomes (L) were concurrent with this observation. However, astrocytic swelling, mitochondrial (M) swelling, endoplasmic reticulum (ER) swelling and fragmentation, and the increase in lysosome (L) number after OGD/R were reduced by HMGB1 inhibition using either HMGB1 shRNA or EP (× 50,000, bar equal to 1 μm, three replicates)

We next examined the effect of inhibiting HMGB1 on cellular swelling in cultured spinal cord astrocytes after OGD/R. Astrocytic swelling was evaluated using astrocytic volume as well as astrocyte morphology and ultrastructure. Treatment with either HMGB1 shRNA or EP (inhibiting HMGB1) was able to significantly block increases in astrocytic cell volume at 6, 12, and 24 h during the reoxygenation process when compared with those of the OGD/R group (*P* < 0.05, Fig. 3a). Morphological and ultrastructural characterization of astrocytes using transmission electron microscopy showed that after OGD/R, spinal cord astrocytes appeared swollen at 6, 12, and 24 h during reoxygenation. The mitochondrial swelling and endoplasmic reticulum swelling and fragmentation and an increase in the number of lysosomes were concurrent with this observation. However, changes to astrocytic morphology and ultrastructure after OGD/R were reduced by inhibiting HMGB1 (Fig. 3b–d). These findings suggest that inhibiting HMGB1 reduces the cellular swelling in cultured spinal cord astrocytes which results from OGD/R.

### Inhibiting HMGB1 decreases expression of HMGB1, AQP4, and TLR4 in cultured spinal cord astrocytes after OGD/R, as well as levels of HMGB1 and IL-6 released into the surrounding medium

**Fig. 4:**
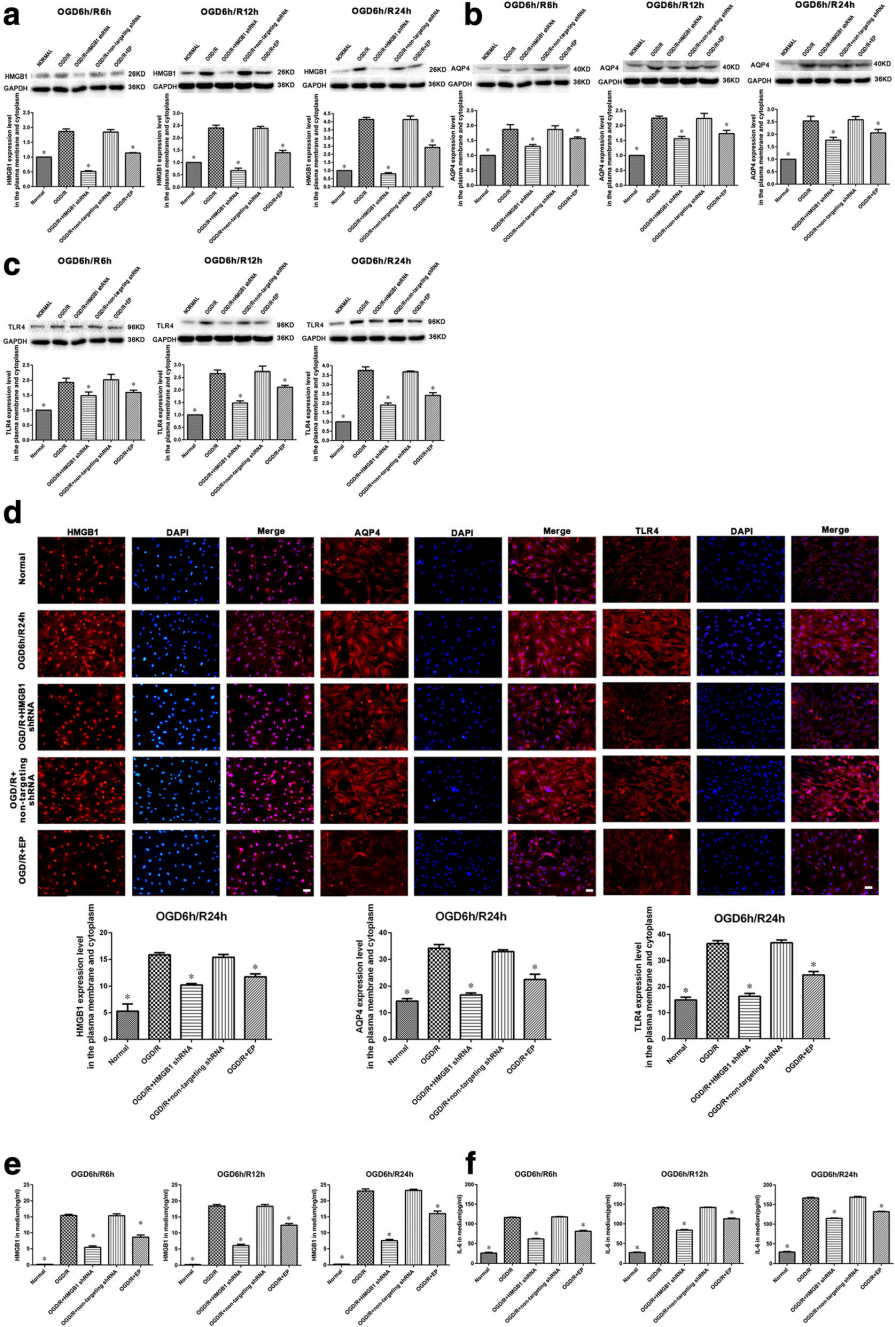
Effects of inhibiting high mobility group box-1 (HMGB1) on HMGB1, aquaporin-4 (AQP4), and toll-like receptor-4 (TLR4) expression in cultured spinal cord astrocytes after oxygen-glucose deprivation/reoxygenation (OGD/R) as well as levels of HMGB1 and interleukin-6 (IL-6) release into the surrounding medium. **a** Inhibiting HMGB1 using either HMGB1 shRNA or ethyl pyruvate (EP) significantly suppressed the increased levels of HMGB1 in both the plasma membrane and cytoplasm of spinal cord astrocytes at 6, 12, and 24 h during reoxygenation after OGD. *P < 0.05 vs. OGD/R group (three replicates). **b** Inhibiting HMGB1 significantly suppressed the increased levels of AQP4 in both the plasma membrane and cytoplasm of spinal cord astrocytes at 6, 12, and 24 h during reoxygenation after OGD. *P < 0.05 vs. OGD/R group (three replicates). **c** Inhibiting HMGB1 significantly suppressed increased levels of TLR4 in both the plasma membrane and cytoplasm of spinal cord astrocytes at 6, 12, and 24 h during reoxygenation after OGD. *P < 0.05 vs. OGD/R group (three replicates). **d** HMGB1, AQP4, and TLR4 immunofluorescence on spinal cord astrocytes at 24 h into the reoxygenation process after OGD showed significantly increased membrane and cytoplasmic levels of HMGB1, AQP4, and TLR4 in the OGD/R group when compared with those in the normal group. These were markedly suppressed in both the OGD/R + HMGB1 shRNA and OGD/R + EP groups (× 200, bar equal to 100 μm). *P < 0.05 vs. OGD/R group (three replicates). **e**, **f** Inhibiting HMGB1 mitigated increases in levels of HMGB1 and IL-6 in the surrounding medium when compared with levels in the OGD/R group at 6, 12, and 24 h during reoxygenation after OGD. *P < 0.05 vs. OGD/R group (three replicates)

Plasma membrane and cytoplasmic extracts were isolated from cultured spinal cord astrocytes from each group after OGD/R and analyzed for HMGB1, AQP4, and TLR4 expression using Western blot. After HMGB1 shRNA administration, the increased levels of HMGB1 were markedly suppressed in the OGD/R + HMGB1 shRNA group at 6, 12, and 24 h during reoxygenation (*P* < 0.05). EP also significantly suppressed the increased levels of HMGB1 in cultured spinal cord astrocytes after OGD/R (*P* < 0.05, Fig. 4a). Western blot analysis revealed that AQP4 protein levels in spinal cord astrocytes were significantly increased in the OGD/R group at 6, 12, and 24 h during reoxygenation (*P* < 0.05), but these increases were significantly attenuated by both HMGB1 shRNA and EP treatments (*P* < 0.05, Fig. 4b). TLR4 protein levels were significantly higher in the OGD/R group than those in the normal group at 6, 12, and 24 h during the reoxygenation process (*P* < 0.05). These levels were reduced by both HMGB1 shRNA and EP administration (*P* < 0.05, Fig. 4c). HMGB1, AQP4, and TLR4 immunofluorescence of spinal cord astrocytes at 24 h during reoxygenation after OGD also revealed significantly increased membrane and cytoplasmic HMGB1, AQP4, and TLR4 levels in the OGD/R group when compared with those in the normal group (*P* < 0.05). Levels were remarkably suppressed in both the OGD/R + HMGB1 shRNA and OGD/R + EP groups (*P* < 0.05, Fig. 4d). Consistent with these results, inhibiting HMGB1 significantly reduced HMGB1, AQP4, and TLR4 expression in cultured spinal cord astrocytes after OGD/R.

ELISA was used to examine HMGB1 levels as well as levels of IL-6 released from cultured spinal cord astrocytes into the surrounding medium of each group after OGD/R. Results showed that inhibiting HMGB1 with either HMGB1 shRNA or EP reduced the increases of HMGB1 and IL-6 in the medium compared with the OGD/R group at 6, 12, and 24 h during reoxygenation (*P* < 0.05, Fig. 4e, f).

### Either HMGB1 or TLR4 inhibition reduces OGD/R-induced spinal cord astrocytic swelling and decreases TLR4, MyD88, and AQP4 upregulation and NF-κB activation, as well as levels of IL-6 released into the surrounding medium

**Fig. 5:**
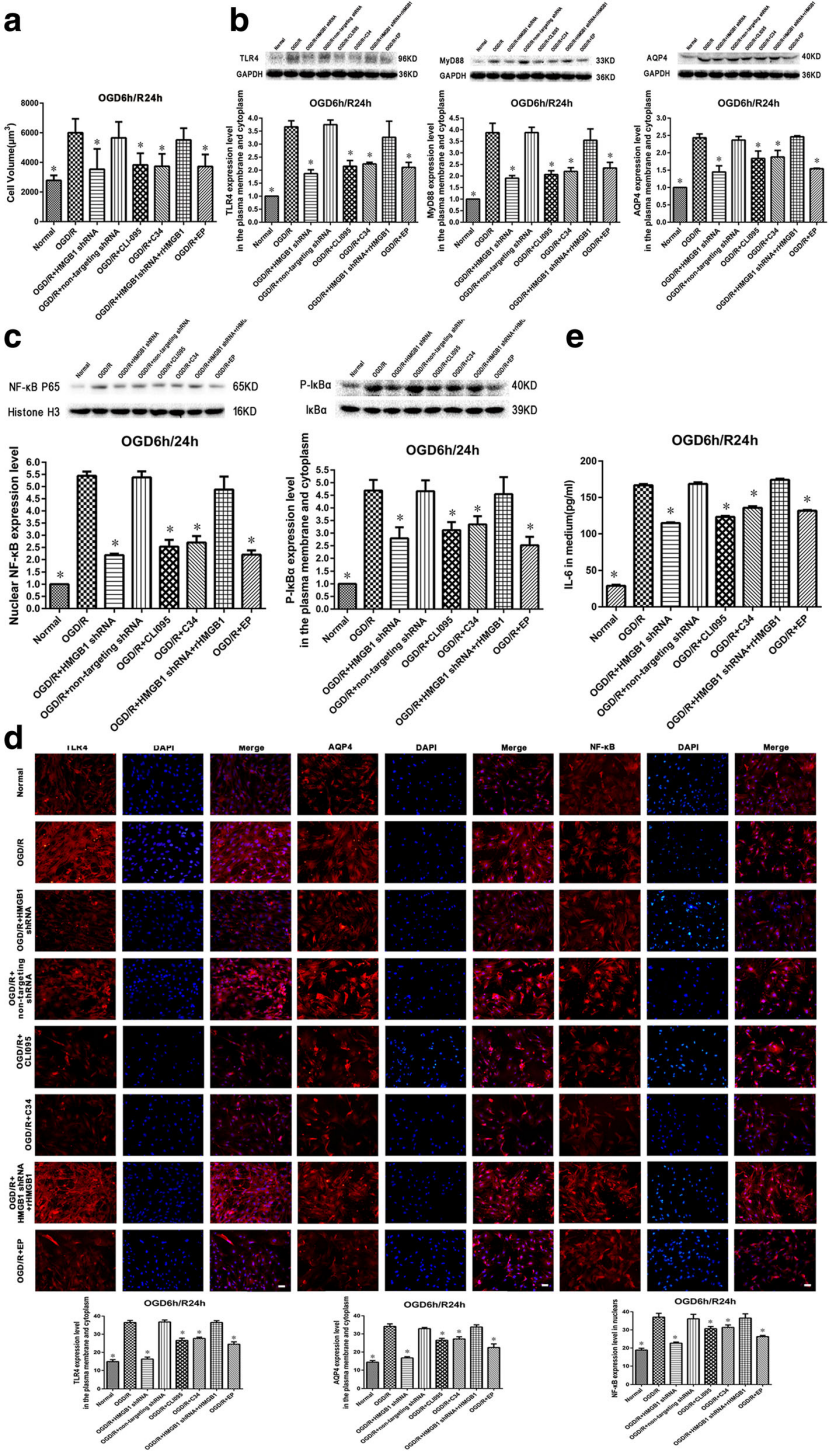
Effects of either inhibiting high mobility group box-1 (HMGB1) or toll-like receptor-4 (TLR4) on oxygen-glucose deprivation/reoxygenation (OGD/R)-induced astrocytic swelling, TLR4, myeloid differentiation primary response gene 88 (MyD88), aquaporin-4 (AQP4) upregulation, and nuclear factor-kappa B (NF-κB) activation as well as levels of interleukin-6 (IL-6) released into the surrounding medium. **a** Inhibiting HMGB1 (using either HMGB1 shRNA or ethyl pyruvate (EP)) or TLR4 (using CLI-095 or C34) significantly reduced the increase in cellular volume of spinal cord astrocytes at 24 h during the reoxygenation process after OGD when compared with those in the OGD/R group. *P < 0.05 vs. OGD/R group (three replicates). **b** Inhibiting HMGB1 or TLR4 significantly suppressed the increased levels of TLR4, MyD88, and AQP4 in both the plasma membrane and cytoplasm of spinal cord astrocytes at 24 h during the reoxygenation process after OGD. *P < 0.05 vs. OGD/R group (three replicates). **c** Inhibiting HMGB1 or TLR4 significantly suppressed the increased nuclear levels of NF-κB and the upregulation of cytoplasmic p-IκBα in spinal cord astrocytes after 24 h of the reoxygenation process after OGD. *P < 0.05 vs. OGD/R group (three replicates). **d** Immunofluorescence results showed that either inhibiting HMGB1 or TLR4 decreased membrane and cytoplasmic TLR4 and AQP4 upregulation and attenuated the increases of nuclear NF-κB when compared with the OGD/R group at 24 h during reoxygenation (× 200, bar equal to 100 μm). *P < 0.05 vs. OGD/R group (three replicates). **e** Inhibiting HMGB1 or TLR4 reduced increased levels of IL-6 in the surrounding medium when compared with those of the OGD/R group after 24 h of the reoxygenation process after OGD. *P < 0.05 vs. OGD/R group (three replicates)

The increase in cell volume of spinal cord astrocytes at 24 h during reoxygenation after OGD was significantly reduced by either inhibiting HMGB1 (using HMGB1 shRNA or EP) or TLR4 (using CLI-095 or C34) when compared with the OGD/R group (*P* < 0.05, Fig. 5a).

Membrane and cytoplasmic TLR4, MyD88, and AQP4 expressions in spinal cord astrocytes were analyzed using Western blot. Results indicated that either inhibiting HMGB1 or TLR4 decreased TLR4, MyD88, and AQP4 upregulation when compared with the OGD/R group at 24 h during reoxygenation (*P* < 0.05, Fig. 5b).

NF-κB activation in spinal cord astrocytes was indicated by nuclear NF-κB levels as well as changes in cytoplasmic p-IκBα expression. Using Western blot, we tested nuclear NF-κB and cytoplasmic p-IκBα levels. Results showed that OGD/R significantly increased both nuclear NF-κB and cytoplasmic p-IκBα (*P* < 0.05), while either inhibiting HMGB1 or TLR4 significantly attenuated the increases of nuclear NF-κB and cytoplasmic p-IκBα when compared with those in the OGD/R group 24 h after reoxygenation (*P* < 0.05, Fig. 5c).

Membrane and cytoplasmic TLR4 and AQP4 expressions in spinal cord astrocytes were analyzed using immunofluorescence. We also tested astrocytic nuclear NF-κB using immunofluorescence. Results showed that either inhibiting HMGB1 or TLR4 decreased membrane and cytoplasmic TLR4 and AQP4 upregulation and attenuated the increases of nuclear NF-κB when compared with the OGD/R group at 24 h during reoxygenation (*P* < 0.05, Fig. 5d).

ELISA was used to examine IL-6 levels in the surrounding medium. Results revealed that either inhibiting HMGB1 or TLR4 reduced IL-6 increases in the medium when compared with the OGD/R group at 24 h during reoxygenation (*P* < 0.05, Fig. 5e).

We also found that rHMGB1, when added into HMGB1 knockdown spinal cord astrocytes, increased astrocytic swelling. It also increased TLR4, MyD88, and AQP4 expression; NF-κB activation; and levels of IL-6 released into the surrounding medium when compared with the OGD/R + HMGB1 shRNA group at 24 h during the reoxygenation process after OGD (*P* < 0.05, Fig. 5a–e).

### NF-κB inhibition reduces OGD/R-induced astrocytic swelling and decreases NF-κB activation and AQP4 upregulation as well as IL-6 released into the surrounding medium

**Fig. 6:**
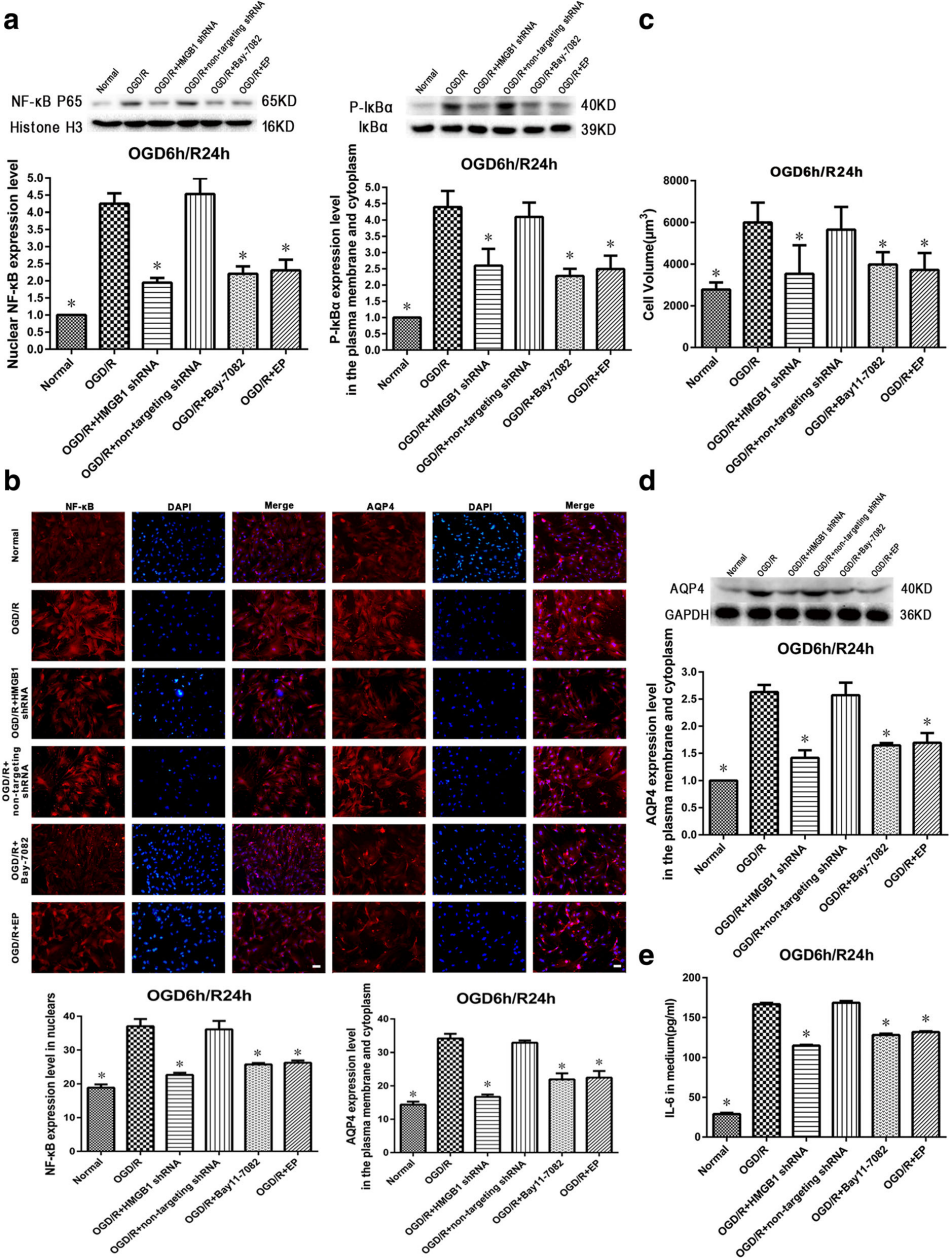
Effects of nuclear factor-kappa B (NF-κB) inhibition on oxygen-glucose deprivation/reoxygenation (OGD/R)-induced astrocytic swelling, NF-κB activation, and aquaporin-4 (AQP4) upregulation, as well as levels of interleukin-6 (IL-6) released into the surrounding medium. **a** NF-κB inhibition (using BAY 11-7082) significantly suppressed the increased nuclear levels of NF-κB and the upregulation of cytoplasmic p-IκBα in spinal cord astrocytes after 24 h of the reoxygenation phase after OGD. *P < 0.05 vs. OGD/R group (three replicates). **b** NF-κB and AQP4 immunofluorescence in spinal cord astrocytes after 24 h of the reoxygenation process after OGD showed significantly increased nuclear levels of NF-κB and membrane and cytoplasmic levels of AQP4 in the OGD/R group. Levels were markedly attenuated in the OGD/R + HMGB1 shRNA, OGD/R + BAY 11-7082, and OGD/R + EP groups (× 200, bar equal to 100 μm). *P < 0.05 vs. OGD/R group (three replicates). **c** NF-κB inhibition significantly reduced the increase in cellular volume of spinal cord astrocytes at 24 h during the reoxygenation process after OGD when compared with those of the OGD/R group. *P < 0.05 vs. OGD/R group (three replicates). **d** NF-κB inhibition significantly suppressed increased AQP4 levels in both the plasma membrane and cytoplasm of spinal cord astrocytes after 24 h of the reoxygenation process after OGD. *P < 0.05 vs. OGD/R group (three replicates). **e** NF-κB inhibition reduced increased levels of IL-6 in the surrounding medium when compared with those of the OGD/R group after 24 h of the reoxygenation process after OGD. *P < 0.05 vs. OGD/R group (three replicates)

The NF-κB inhibitor, BAY 11-7082, was used in this experiment. Nuclear NF-κB and cytoplasmic p-IκBα were determined using Western blot, and results indicated NF-κB activation in spinal cord astrocytes. NF-κB inhibition significantly attenuated increases in nuclear NF-κB and cytoplasmic p-IκBα when compared with the OGD/R group at 24 h during reoxygenation (*P* < 0.05, Fig. 6a). Immunofluorescence for NF-κB and AQP4 at 24 h during reoxygenation after OGD also showed that nuclear levels of NF-κB and membrane and cytoplasmic levels of AQP4 were significantly increased in the OGD/R group when compared with the normal group (*P* < 0.05). Levels were remarkably attenuated in the OGD/R + HMGB1 shRNA, OGD/R + BAY 11-7082, and OGD/R + EP groups (*P* < 0.05, Fig. 6b).

The increase in cellular volume of spinal cord astrocytes at 24 h during reoxygenation after OGD was significantly reduced by NF-κB inhibition when compared with the OGD/R group (*P* < 0.05, Fig. 6c).

According to our Western blot data for AQP4 expression in spinal cord astrocytes, NF-κB inhibition decreased AQP4 upregulation when compared with the OGD/R group at 24 h during the reoxygenation process after OGD (*P* < 0.05, Fig. 6d).

ELISA results showed that NF-κB inhibition reduced IL-6 increases in the medium when compared with the OGD/R group at 24 h during the reoxygenation process after OGD (*P* < 0.05, Fig. 6e).

### Effects of rHMGB1 on the regulation of AQP4 expression in cultured spinal cord astrocytes

**Fig. 7:**
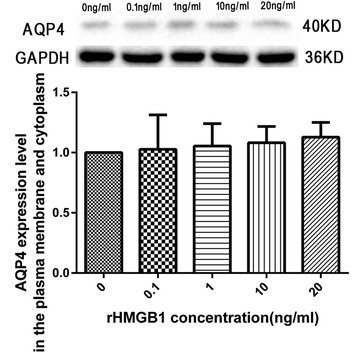
Effects of recombinant HMGB1 (rHMGB1) on aquaporin-4 (AQP4) expression in cultured spinal cord astrocytes. Incubation of cultured spinal cord astrocytes with rHMGB1 (0, 0.1, 1, 10, and 20 ng/ml) for 24 h did not induce dose-dependent increases in the membrane and cytoplasmic AQP4 expression (three replicates)

Western blot analysis revealed that incubation of cultured spinal cord astrocytes with rHMGB1 (0, 0.1, 1, 10, and 20 ng/ml) for 24 h did not induce dose-dependent increases in AQP4 expression (*P* > 0.05, Fig. 7).

### IL-6 mediates AQP4 expression in cultured spinal cord astrocytes

**Fig. 8:**
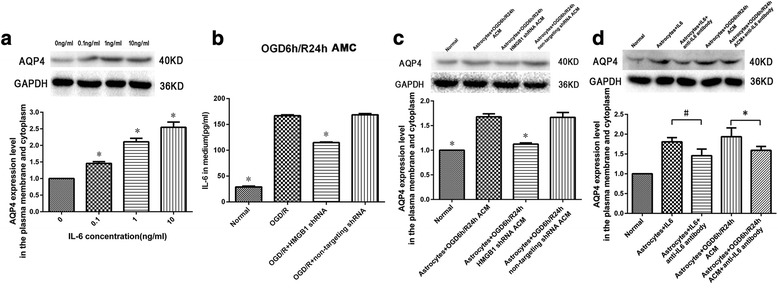
Effects of interleukin-6 (IL-6) on aquaporin-4 (AQP4) expression in cultured spinal cord astrocytes. **a** Spinal cord astrocytes were exposed to exogenous IL-6 at 0, 0.1, 1, or 10 ng/ml. After 24 h exposure, the membrane and cytoplasmic AQP4 expression in spinal cord astrocytes were markedly increased in the IL-6 0.1 ng/ml group, IL-6 1 ng/ml group, and IL-6 10 ng/ml group. *P < 0.05 vs. 0 ng/ml group (three replicates). **b** IL-6 levels increased in the surrounding medium of the OGD/R group after 24 h of the reoxygenation process after OGD. In comparison, this increase was significantly reduced in the OGD/R + HMGB1 shRNA group. *P < 0.05 vs. OGD/R group (three replicates). **c** The effects of astrocyte conditioned medium (ACM) on AQP4 expression in cultured spinal cord astrocytes. Twenty-four hours exposure of spinal cord astrocytes to the ACM obtained from the OGD/R group significantly increased the membrane and cytoplasmic AQP4 expression when compared with astrocytes incubated with the ACM obtained from the OGD/R + HMGB1 shRNA group. *P < 0.05 vs. astrocytes + OGD6h/R24h ACM group (three replicates). **d** Western blot analysis showed that the neutralizing anti-rat-IL-6 antibody could significantly reverse the upregulation effect of exogenous IL-6 or OGD/R ACM containing increased IL-6 on AQP4 expression in cultured spinal cord astrocytes. #*P* < 0.05 vs. astrocytes + IL-6 group; *P < 0.05 vs. astrocytes + OGD6h/R24h ACM group (three replicates)

After exposure of spinal cord astrocytes to exogenous IL-6 at 0.1, 1, or 10 ng/ml for 24 h, the membrane and cytoplasmic AQP4 expressions in spinal cord astrocytes were markedly increased in IL-6 0.1 ng/ml, IL-6 1 ng/ml, and IL-6 10 ng/ml groups when compared with the IL-6 0 ng/ml group (*P* < 0.05, Fig. 8a).

IL-6 levels increased in the medium of the OGD/R group at 24 h during reoxygenation after OGD (166.67 ± 1.76 pg/ml). This increase was reduced in the OGD/R + HMGB1 shRNA group (*P* < 0.05, Fig. 8b). Western blot results showed that exposure of the cultured spinal cord astrocytes to ACM from the OGD/R group for 24 h significantly increased AQP4 expression when compared with ACM from the OGD/R + HMGB1 shRNA group (*P* < 0.05, Fig. 8c).

Furthermore, the upregulation effect of exogenous IL-6 or OGD/R ACM containing increased IL-6 on AQP4 expression in cultured spinal cord astrocytes could be significantly reversed by the neutralizing anti-rat-IL-6 antibody (*P* < 0.05, Fig. 8d). Together, these data implicate IL-6 as a factor promoting AQP4 expression in spinal cord astrocytes after OGD/R.

## Discussion

Our studies demonstrated that cultured spinal cord astrocytes exposed to OGD/R had increased cell swelling as well as increased astrocytic expressions of HMGB1 and AQP4. In addition, OGD/R resulted in increased HMGB1 and IL-6 levels in the surrounding medium. Inhibiting HMGB1 after OGD/R in vitro using either HMGB1 shRNA or EP reduced astrocytic swelling, repressed AQP4 overexpression, and decreased HMGB1 and IL-6 levels in the surrounding medium. Further experiments showed that this reduction in spinal cord astrocyte swelling and AQP4 expression through HMGB1 inhibition might be associated with inhibition of the activation of the HMGB1/TLR4/MyD88/NF-κB signaling pathway. This would result in IL-6 production and subsequent AQP4 upregulation following OGD/R in cultured spinal cord astrocytes.

HMGB1 is a non-histone DNA-binding protein that plays a role in transcriptional regulation in cells. Critically, it also serves as a cytokine-like mediator of inflammation outside cells [41]. In the central nervous system (CNS), extracellular HMGB1 induces neuroinflammation and could be an early trigger for proinflammatory activation, including tissue edema [17, 27, 28, 32]. After spinal cord injury (SCI) in humans, plasma HMGB1 levels in persons with acute SCI are significantly higher than in uninjured persons [15]. HMGB1 can be actively secreted from reactive microglia and astrocytes as well as passively released from necrotic cells after pathogenic and/or tissue injury in the CNS. Of these, reactive astrocytes are one of the primary sources of HMGB1 [15, 17–21].

In this study, we found that both HMGB1 expression in cultured spinal cord astrocytes and HMGB1 levels in the surrounding medium were significantly increased in vitro after OGD/R. Extracellular HMGB1 is known to bind and signal through cell surface receptors including TLR4, TLR2, and RAGE in immune-competent cells, neurons, and astrocytes [17, 22, 23, 32]. Downstream effects are also wide-ranging, and one of the cascades is NF-κB activation. This induces the production of proinflammatory cytokines such as TNF-α, IL-1β, and IL-6 as well as the general initiation and regulation of the inflammatory response [24–28].

Spinal cord edema occurring after SCI has been associated with poor neurological outcomes [1, 3, 42]. The mechanisms for spinal cord edema formation are comparable to those in the brain, which include cytotoxic and vasogenic edema [43]. Cytotoxic edema predominates in the initial phase after SCI and involves the accumulation of excess fluid in intracellular compartments and disturbances in cell volume regulation [8, 9, 43, 44]. Astrocytic swelling represents the major component of cytotoxic edema in the CNS [7, 45]. The mechanisms of astrocytic swelling in various neurological conditions remain unclear, but AQP4 has emerged as a prime candidate. AQP4 is not only localized in astrocytic end-feet, but also dispersed in the cytoplasm of reactive astrocytes [11–13, 44, 46]. AQP4 overexpression might contribute to increased water into astrocytes, thereby allowing for the development of cytotoxic edema and the exacerbation of spinal cord edema [1, 8, 13, 14, 42, 43].

Here, we cultured astrocytes originating from rat spinal cord. These astrocytes were then subjected to OGD/R to establish a cell model of SCI. Astrocytic swelling as well as AQP4 expression and regulation in astrocytes were subsequently studied. Our results showed that cellular volume and AQP4 protein levels in astrocytes both increased and peaked at 24 h after OGD/R. These results confirmed previous findings showing that AQP4 upregulation was closely associated with the development of astrocytic swelling [44, 46, 47].

In addition, our study measured astrocytic cell volume using a Live Cell Imaging System [39, 40]. We stained cultured astrocytes and suspended single cells without injury. Since the living, suspended single astrocytes were similar in shape to a sphere, we obtained the biggest Z-slice image of the cell by overlapping many slices in the stack. This allowed us more exact measurements and calculations, resulting in more accurate astrocytic cellular volumes.

RNA interference technology is a very powerful tool that allows for acute, transient knockdown of selected proteins. In the CNS, HMGB1 protein expression has been effectively knocked down using RNA interference approaches [20, 21, 48]. In this study, we efficiently delivered shRNA using a lentiviral vector to spinal cord astrocytes at concentrations that were not toxic. This HMGB1-specific shRNA resulted in decreased HMGB1 protein levels in both plasma membrane and cytoplasmic extracts of control values. In addition, EP was also used to inhibit HMGB1 expression [34]. HMGB1 inhibition resulted in reduced astrocytic swelling and attenuated astrocytic AQP4 overexpression—both of which increased in cultured spinal cord astrocytes after OGD/R. Studies have shown that HMGB1 is important for AQP4 upregulation in brain astrocytes and subsequent formation of brain edema after injury [27, 28]. Moreover, Jian et al. [49] reported that probenecid reduced brain edema and astrocytic swelling by inhibiting HMGB1 release. This alleviated cerebral inflammation and attenuated AQP4 in both an in vivo brain injury model and an in vitro brain astrocyte OGD/R model. Some reports have also shown that decreases in AQP4 were more closely associated with reductions in astrocytic swelling [40, 47, 50].

We further studied the possible mechanisms behind the reduction of spinal cord astrocytic swelling and AQP4 overexpression by inhibiting HMGB1. After OGD/R, administration of either HMGB1 shRNA or EP markedly reduced spinal cord astrocytic swelling and AQP4 overexpression. They also significantly suppressed plasma and cytoplasmic upregulations of HMGB1, TLR4, and MyD88; nuclear levels of NF-κB; and IL-6 levels in the surrounding medium. These findings suggest one possible mechanism in this complex, interconnecting signaling network. TLR4 is one of the receptors in the CNS on which HMGB1 signaling depends [34, 51]. TLR4 is expressed in the spinal cord tissue as well as cultured astrocytes [16, 36, 52]. Crucially, the interaction between HMGB1 and TLR4 could trigger several signaling pathways. To this end, TLR4 signals through adaptive signaling pathways (e.g., MyD88), leading to activation and translocation of NF-κB. This subsequently produces proinflammatory cytokines such as TNF-α, IL-1β, and IL-6 or adjusts effector protein levels [4, 25, 29, 34]. To confirm the role of TLR4, the TLR4 inhibitors C34 and CLI-095 were used in this study. Our results showed that these TLR4 inhibitors reduced spinal cord astrocytic swelling and suppressed TLR4, MyD88, and AQP4 overexpression. Moreover, TLR4 inhibition attenuated NF-κB activation and decreased IL-6 levels in the surrounding medium when compared with the OGD/R group. Consistent with our view and also as reported, TLR4 inhibition has been shown to reduce AQP4 expression and astrocytic swelling in other injury and disease states in the CNS [27, 29]. To further examine this connection, rHMGB1 was added back into the medium of spinal cord astrocytes with HMGB1 knockdown. Critically, this resulted in cultured spinal cord astrocytic swelling, indicating that the added HMGB interacted with TLR4 and, in other words, that TLR4 signaling activation and AQP4 overexpression occur after OGD/R.

NF-κB plays a major role in the inflammatory response since many genes associated with inflammation are regulated by NF-κB. It has been shown that the interaction between HMGB1 and TLR4 could result in IκBα phosphorylation, IκB degradation, translocation of NF-κB to the nucleus, and subsequent stimulation of cytokine production [53, 54]. Increasing evidence also suggests that NF-κB activation induces cell swelling and AQP4 overexpression in astrocytes exposed to trauma or the neurotoxin ammonia and, furthermore, that the inhibition of such activation significantly reduces astrocyte swelling and AQP4 overexpression [30, 44, 55]. Consistent with these findings, we found that after OGD/R, spinal cord astrocytes increased cellular swelling and AQP4, HMGB1, and TLR4 expression. In addition, phosphorylation of IκBα was increased and NF-κB was activated. Inhibition of either HMGB1 or TLR4 reduced astrocytic swelling, AQP4 overexpression, and activation of NF-κB. Moreover, exposing spinal cord astrocytes to OGD/R that were treated with BAY 11-7082 (an inhibitor of NF-κ B) showed a lesser degree of cell swelling and lower expression of AQP4 when compared to astrocytes subjected only to OGD/R and no treatment. Taken together, these results strongly implicate HMGB1/TLR4 binding as an activator of the NF-κB signaling pathway. This activation results in spinal cord astrocytic swelling.

IL-6 is an important proinflammatory cytokine that can be found after SCI and can be produced by the HMGB1/TLR4/NF-κB signaling pathway [4, 26, 32]. Laird et al. [27] reported that HMGB1 could initiate IL-6 release in a TLR4-dependent mechanism and that IL-6 increased AQP4 expression and cellular swelling of brain astrocytes. Rama Rao et al. [56] reported that proinflammatory cytokines—including IL-6—induced significant cellular swelling in cultured astrocytes. In our study, IL-6 levels increased in the surrounding medium of cultured spinal cord astrocytes after OGD/R, which was accompanied by astrocytic swelling and AQP4 overexpression. All of these effects were suppressed by HMGB1, TLR4, or NF-κB inhibition. Incubation of cultured spinal cord astrocytes with exogenous IL-6 induced increased AQP4 expression. Furthermore, exposure of cultured spinal cord astrocytes to the ACM from the OGD/R group meant exposure to increased IL-6 levels. This exposure resulted in significantly increased astrocytic AQP4 expression, as compared with ACM incubation from the OGD/R + HMGB1 shRNA group. The upregulation effect of exogenous IL-6 or OGD/R ACM on AQP4 expression could be significantly reversed by the neutralizing anti-rat-IL-6 antibody. However, incubation of cultured spinal cord astrocytes with only rHMGB1 did not result in increased AQP4 expression. As a result, we hypothesized that HMGB1 regulated AQP4 expression in cultured spinal cord astrocytes after OGD/R via an IL-6-dependent mechanism. In light of our results, we speculated that inhibiting HMGB1 using either HMGB1 shRNA or EP after OGD/R would reduce HMGB1 expression. In turn, this would further decrease the interactions between HMGB1 and its one receptor, TLR4. This decrease in interaction might subsequently affect the TLR4/MyD88/NF-κB signaling pathway that produces proinflammatory cytokines (e.g., IL-6). Consequently, there would be a comprehensive impact on the reduction in AQP4 expression and spinal cord astrocytic swelling.

It should be noted that there are some limitations to this study. First, there are several different cell types in the spinal cord, all of which exert distinct functions and interact with each other during the inflammation process after SCI. Given this, both microglia and endothelial cells play important roles during the development of inflammation and edema after SCI. In the present study, we only observed the effects of inhibiting astrocyte HMGB1 on astrocytic swelling itself. Further in vitro and in vivo studies should be conducted in mixed astrocyte and other spinal cord cell co-culture systems. Second, other receptors of HMGB1, such as TLR2 and RAGE, and other signaling pathways that are involved in HMGB1 intracellular signaling were not investigated. These additional receptors and signaling pathways will need to be targeted in future investigations. Third, many aquaporins and ion channel proteins are involved in the formation of astrocytic swelling. It remains possible that the effects of HMGB1 on the regulation of these proteins may be important to the continued evolution of astrocytic swelling. Further studies will be required to address these questions.

## Conclusions

In summary, HMGB1 upregulates AQP4 expression and promotes cell swelling in spinal cord astrocytes in vitro after OGD/R. These effects occur through HMGB1/TLR4/MyD88/NF-κB signaling and in an IL-6-dependent manner. We found that inhibiting HMGB1 reduced cultured spinal cord astrocytic swelling after OGD/R, which was associated with downregulated astrocytic AQP4 expression. HMGB1 plays a critical role in this process and is a promising, new target for future studies as well as treatment plans for spinal cord astrocytic swelling and tissue edema after SCI.

## Abbreviations

ACM: Astrocyte conditioned media
AQP4: Aquaporin-4
CNS: Central nervous system
Dil: 1,1′-dioctadecyl-3,3,3′,3′-tetramethylindocarbocyanine perchlorate
DMEM: Dulbecco’s modified Eagle medium
ECL: Enhanced chemiluminescence
ELISA: Enzyme-linked immunosorbent assay
EP: Ethyl pyruvate
FBS: Fetal bovine serum
HMGB1: High mobility group box-1
IL-1β: Interleukin-1β
IL-6: Interleukin-6
MOI: Multiplicity of infection
MyD88: Myeloid differentiation primary response gene 88
NF-κB: Nuclear factor-kappa B
OGD/R: Oxygen-glucose deprivation/reoxygenation
PBS: Phosphate-buffered saline
PBST: PBS containing Tween-20
PMSF: Phenylmethanesulfonyl fluoride
PND: Post-natal day
RAGE: Advanced glycation end product
rHMGB1: Recombinant HMGB1
SCI: Spinal cord injury
TLR2: Toll-like receptor 2
TLR4: Toll-like receptor 4
